# Network localization of brain functional effects of ketamine treatment for major depression

**DOI:** 10.1192/j.eurpsy.2026.10164

**Published:** 2026-02-13

**Authors:** Haining Ma, Huaigui Liu, Wenwei Zhang, Xufeng Zhao, Dan Zhang, Kaijie An, Yinfeng Qian, Jiajia Zhu

**Affiliations:** 1 Department of Radiology, The First Affiliated Hospital of Anhui Medical University, Hefei 230022, China; 2 Research Center of Clinical Medical Imaging, Anhui Province, Hefei 230032, China; 3 Anhui Provincial Key Laboratory for Brain Bank Construction and Resource Utilization, Hefei 230032, China; 4 Department of Radiology, Tianjin Key Laboratory of Functional Imaging & Tianjin Institute of Radiology, Tianjin Medical University General Hospital, Tianjin 300052, China

**Keywords:** brain network, depression, functional connectivity, ketamine, MRI

## Abstract

**Background:**

Considerable effort has been devoted to investigate the neuroimaging correlates and predictors of antidepressant response to ketamine, yet inconsistency in the location and nature of the regional brain effects makes it difficult to unify this research. Despite the revolutionary notion that psychiatric therapeutics show network-level brain representations, investigations into network localization of brain functional effects of ketamine treatment are still lacking.

**Methods:**

We initially identified the locations of longitudinal brain functional alterations (increase and decrease separately) induced by ketamine treatment from 16 published studies with 508 depressed patients. By integrating these affected brain locations with large-scale functional MRI datasets from 1113 healthy and 255 depressed individuals, we then leveraged a novel functional connectivity network mapping approach to construct ketamine-induced hyper-functional and hypo-functional networks respectively.

**Results:**

The hyper-functional network mainly involved the subcortical (caudate nucleus and thalamus) and default (medial prefrontal cortex) networks, while its hypo-functional counterpart predominantly implicated the limbic (temporal pole), subcortical (hippocampus and amygdala), and default (lateral temporal cortex) networks.

**Conclusion:**

Our findings may shed light on the neurobiological effects of ketamine from a network perspective, which might represent a crucial step toward fostering the clinical application of ketamine in antidepressant treatment.

## Introduction

Treatment for depressed patients with major depressive disorder (MDD) or bipolar disorder (BD) commonly involves pharmacological therapy with antidepressant medications [1, 2]. Despite the proven moderate efficacy, conventional monoamine-based antidepressants typically suffer from several limitations, including low rates of remission, the failure of most treated individuals to achieve full symptomatic and functional recovery, a similar mechanism of action (largely via enhancement of monoaminergic neurotransmission), and a delayed onset of action requiring weeks to achieve significant therapeutic effects [1, 3, 4]. The discovery of a rapid antidepressant effect from the N-methyl-D-aspartate receptor (NMDAR) antagonist ketamine can potentially overcome some of the limitations, since mounting evidence supports the rapid antidepressant efficacy of ketamine in treatment-resistant depression, with an onset of action within hours [4–6].

The study of brain-based correlates and predictors of antidepressant response to ketamine has been the subject of intensive investigation in neuroscience and psychiatry, providing important insights into the neurobiological therapeutic mechanisms of ketamine. Experimental animal research has documented that ketamine exerts its antidepressant effect via blocking NMDAR-dependent bursting activity in the lateral habenula to disinhibit downstream monoaminergic reward centers [7–9]. In parallel, a large number of human neuroimaging studies have attempted to identify brain biomarkers associated with ketamine administration, but mostly with small sample sizes and substantial methodological heterogeneity that have led to mixed and inconclusive results [10–14], leaving us with a poor understanding of the ketamine-induced brain changes in humans.

The majority of neuroimaging studies have focused on regional brain abnormalities in relation to a psychiatric illness or a therapeutic intervention. However, it is now increasingly evident that psychiatric disorders localize better to distributed brain networks than to individual anatomical regions [15–17], due to the fact that neuropathological processes do not act in isolation but rather are interconnected via distributed networks. The advanced network-based conceptualization of psychiatric disorders has recently given rise to the revolutionary notion that psychiatric therapeutics also show network-level brain representations and yield clinical benefits in a network-dependent manner [18–20]. To achieve network localization, investigators have developed a novel and well-validated functional connectivity network mapping (FCNM) approach [21–24] that can map brain locations associated with a disease or a treatment to a common network by use of the human brain functional connectome. This network-based framework has enjoyed significant success in localizing psychiatric disorders [25–30] and therapeutic interventions [31, 32] to specific brain networks, improving our understanding of disease and treatment mechanisms from a network perspective. Despite these previous efforts, studies investigating the network localization of brain functional alterations induced by ketamine administration in depressed patients are still lacking.

**Figure 1.**
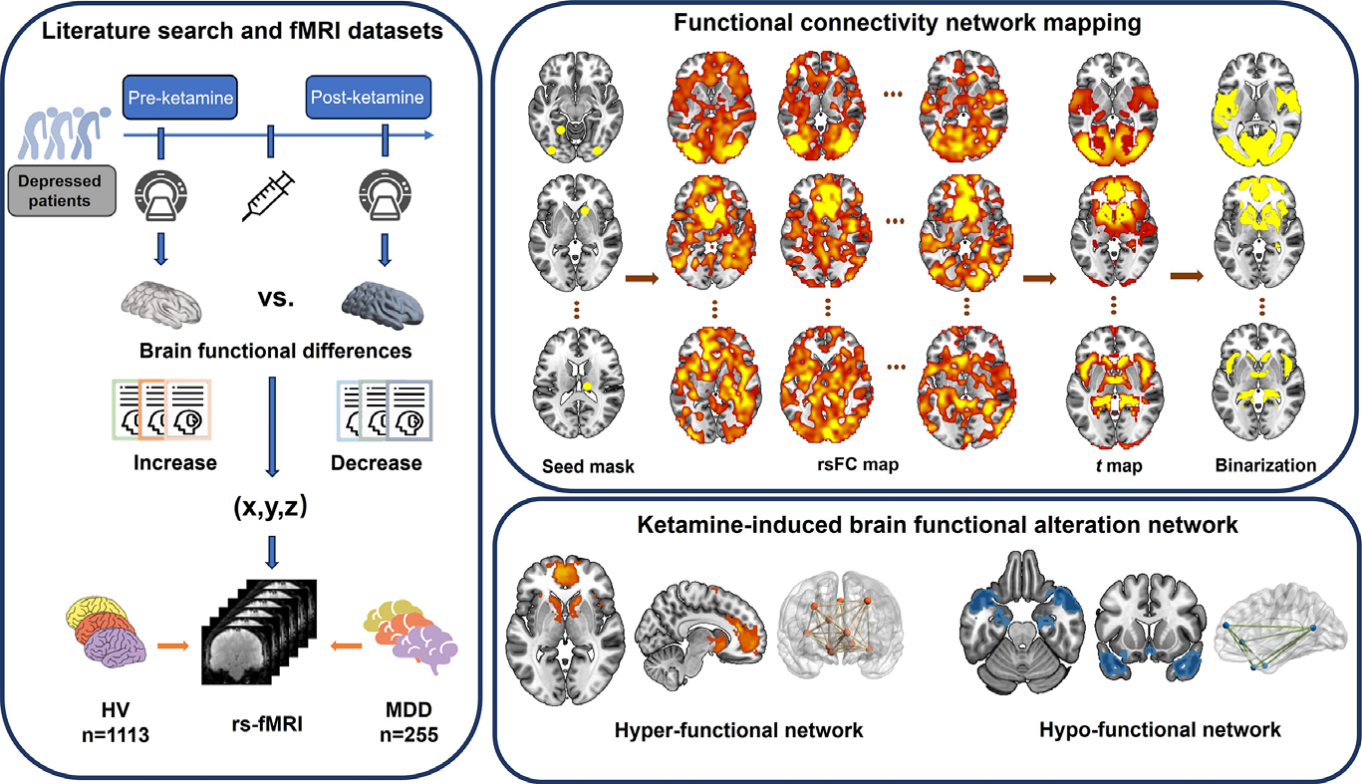
Study design and analysis pipeline. We initially synthesized published literature to identify the locations of longitudinal brain functional alterations (increase and decrease separately) induced by ketamine treatment in depressed patients. By integrating these affected brain locations with large-scale resting-state fMRI datasets (the HV and MDD datasets), we then leveraged the FCNM approach to construct ketamine-induced brain functional alteration networks (hyper-functional and hypo-functional networks respectively). Specifically, spheres centered at each coordinate of a contrast were firstly created and merged together to yield a contrast-specific combined seed mask. Second, in combination with the resting-state fMRI data, we calculated a contrast seed-to-whole brain rsFC map for each subject. Third, the subject-level rsFC maps were entered into a voxel-wise one-sample *t* test to identify brain areas functionally connected to each contrast seed. Fourth, the resultant group-level *t* maps were thresholded and binarized. Finally, the binarized maps were overlaid to produce 2 network probability maps, which were thresholded at 50% to yield ketamine-induced hyper-functional and hypo-functional networks respectively. Abbreviations: HV, healthy volunteer; MDD, major depressive disorder; rs-fMRI, resting-state functional magnetic resonance imaging; rsFC, resting-state functional connectivity.

To address this gap, we initially synthesized published literature to identify the locations of longitudinal brain functional alterations (increase and decrease separately) induced by ketamine treatment in depressed patients. By integrating these affected brain locations with large-scale resting-state functional magnetic resonance imaging (fMRI) datasets, we then leveraged the FCNM approach to construct ketamine-induced brain functional alteration networks (hyper-functional and hypo-functional networks, respectively). A schematic overview of the study design and analysis pipeline is provided in Figure 1.

## Methods and materials

### Study selection

We identified relevant studies examining longitudinal brain functional alterations following ketamine administration (i.e., post- versus pre-ketamine) in depressed patients. Twelve studies published before April 16, 2022, were initially selected from a comprehensive systematic review [12]. To search for additional qualified studies, we then performed a systematic literature search in PubMed and Web of Science to identify relevant studies published from April 16, 2022 to January 2, 2024. The following combination of search terms were used: (“ketamine”) AND (“MRI” OR “fMRI” OR “magnetic resonance imaging” OR “PET” OR “positron emission tomography” OR “SPECT” OR “single photon emission computed tomography” OR “ASL” OR “arterial spin labeling” OR “neuroimaging” OR “FC” OR “functional connectivity” OR “ReHo” OR “regional homogeneity” OR “ALFF” OR “fALFF” OR “amplitude of low frequency fluctuation*” OR “CBF” OR “blood flow” OR “glucose metabolism” OR “VBM” OR “voxel-based morphometry” OR “DBM” OR “deformation-based morphometry” OR “GMV” OR “gray matter” OR “gray matter” OR “EEG” OR “electroencephalogram” OR “MEG” or “magnetoencephalography”). A flow diagram of the study selection process is shown in Supplementary Figure S1. This protocol was registered on PROSPERO (https://www.crd.york.ac.uk/PROSPERO/; registration number: CRD42024565435). It is notable that our initial search terms included structural MRI, EEG, and MEG to ensure a comprehensive review. However, during the full-text screening, we found that the available structural and electrophysiological studies did not meet our inclusion criteria or lacked compatible coordinate data. Therefore, they were excluded from the final analysis. Because a single study may contain multiple contrasts (i.e., within-subject comparisons between pre- and post-ketamine in different brain functional measures), we focused our analyses on contrasts rather than studies. In cases where multiple publications or contrasts utilized the same group of participants, we assessed the neuroimaging measures used. If the measures reflect distinct brain functional properties and contain different neurophysiological information, we treated them as independent inputs. Coordinates of peak voxels of significant clusters reported in each contrast were extracted, with coordinates in Talairach space converted to Montreal Neurological Institute (MNI) space.

To ensure rigor, we designed a specific quality assessment framework based on the Newcastle-Ottawa Scale (NOS), adapted for this study’s context. We evaluated all included studies across four domains: 1) Participants & Selection, 2) Study Design & Treatment Protocol, 3) Imaging Quality (Modality Agnostic), and 4) Statistical Rigor. Based on these scores, studies were classified as: High Quality (14–18 points): no significant shortcomings in design, administration, imaging, or statistics; Moderate Quality (9–13 points): minor defects (e.g., single-blind design or imprecise scan timing) or limitations addressed in the discussion; Low Quality (0–8 points): major defects (e.g., no control, no correction, irregular administration). The detailed scoring criteria and results are provided in Tables S1 and S2 in the Supplementary Materials.

### Brain functional connectome datasets

The resting-state fMRI datasets used for constructing the brain functional connectome included a healthy volunteer (HV) dataset and an MDD dataset. The HV dataset included 1113 healthy adults of Han Chinese descent (643 female, mean 32.66 ± 12.78 years, 1110 right-handed), who were enrolled from local universities and communities through poster advertisements. The exclusion criteria included neuropsychiatric or severe somatic disorders, a history of head injury with consciousness loss, MRI contraindications, or a family history of psychiatric diseases among first-degree relatives. The MDD dataset included 255 MDD individuals of Han Chinese (161 female, mean 40.99 ± 12.67 years, 252 right-handedness), who were recruited consecutively from Affiliated Psychological Hospital of Anhui Medical University. Following the International Classification of Diseases (ICD-10), two well-trained clinical psychiatrists confirmed the diagnosis of depression. The exclusion criteria included 1) the presence of other psychiatric disorders such as substance-induced (e.g., drug or alcohol) mood disorder, bipolar disorder, anxiety disorder, schizophrenia, substance abuse or addiction; 2) a history of severe physical or neurological disease; 3) a history of head injury with loss of consciousness; 4) contraindications for MRI such as pregnancy. This study was approved by the ethics committee of The First Affiliated Hospital of Anhui Medical University. Written informed consent was obtained from all participants after being given a complete description of the study. Demographic and clinical information of the two datasets is provided in Table S3 in the Supplementary Materials.

### fMRI data acquisition and preprocessing

MRI data of the two datasets were collected on a 3.0-Tesla MR system (Discovery MR750w, General Electric, Milwaukee, WI, USA) with the same imaging protocol. During the scans, all participants were instructed to keep their eyes closed, relax but not fall asleep, think of nothing in particular, and move as little as possible. High-resolution 3D T1-weighted structural images were acquired by employing a brain volume (BRAVO) sequence with the following parameters: repetition time (TR) = 8.5 ms; echo time (TE) = 3.2 ms; inversion time (Tl) = 450 ms; flip angle (FA) = 12 °; field of view (FOV) = 256 mm × 256 mm; matrix = 256 × 256; slice thickness = 1 mm; no gap; 188 sagittal slices; acquisition time = 296 s. Resting-state blood-oxygen-level-dependent (BOLD) fMRI data were acquired using a gradient-echo single-shot echo planar imaging (GRE-SS-EPI) sequence with the following parameters: TR = 2000 ms; TE = 30 ms; FA = 90 °; FOV = 220 mm × 220 mm; matrix = 64 × 64; slice thickness = 3 mm; slice gap = 1 mm; 35 interleaved axial slices; 185 volumes; acquisition time = 370 s. Participants with poor image quality (e.g., visible artifacts) or incomplete brain coverage were excluded.

Resting-state fMRI data were preprocessed using Statistical Parametric Mapping (SPM12, http://www.fil.ion.ucl.ac.uk/spm) and Data Processing & Analysis for Brain Imaging (DPABI, http://rfmri.org/dpabi) [33]. The first 10 volumes for each participant were discarded to allow the signal to reach equilibrium and the participants to adapt to the scanning noise. The remaining volumes were corrected for the acquisition time delay between slices. Then, realignment was performed to correct the motion between time points. Head motion parameters were computed by estimating the translation in each direction and the angular rotation on each axis for each volume. All participants’ BOLD data were within the defined motion thresholds (i.e., translational or rotational motion parameters less than 2 mm or 2 ° for healthy subjects and less than 3 mm or 3 ° for MDD individuals). We also calculated frame-wise displacement (FD), which indexes the volume-to-volume changes in head position. Several nuisance covariates (the linear drift, the estimated motion parameters based on the Friston-24 model, the spike volumes with FD > 0.5 mm, the global signal, the white matter signal, and the cerebrospinal fluid signal) were regressed out from the data. Next, the datasets were band-pass filtered using a frequency range of 0.01–0.1 Hz. In the normalization step, individual structural images were first co-registered with the mean functional images; the transformed structural images were then segmented and normalized to MNI space using a high-level nonlinear warping algorithm, i.e., the diffeomorphic anatomical registration through exponentiated Lie algebra technique [34]. Then, each filtered functional volume was spatially normalized to MNI space using the deformation parameters estimated during the above step and resampled into a 3-mm isotropic voxel. Finally, all functional data were spatially smoothed with a Gaussian kernel of 6 × 6 × 6 mm^3^ full-width at half maximum.

### Functional connectivity network mapping

The FCNM approach was used to construct ketamine-induced brain functional alteration networks based on the extracted coordinates of pre- to post-ketamine changes (increase and decrease separately) in brain function (Figure 1). First, 4-mm radius spheres centered at each coordinate of a contrast were created and merged together to yield a contrast-specific combined seed mask (henceforth referred to as the contrast seed). Second, in combination with the preprocessed resting-state fMRI data of the HV and MDD datasets, we calculated a contrast seed-to-whole brain functional connectivity (FC) map for each subject, by computing Pearson’s correlation coefficients between time courses of the contrast seed and each voxel within the whole brain, followed by Fisher’s *Z*-transformation to improve normality. Third, the subject-level FC maps were entered into a voxel-wise one-sample *t* test to identify brain areas functionally connected to each contrast seed. Only positive FC was considered because the biological relevance of negative FC remains controversial [35, 36]. Fourth, the resultant group-level *t* maps were thresholded at *P* < 0.05 adjusted for multiple comparisons and then binarized. Note that a voxel-level family-wise error correction was used for the HV dataset, while a less stringent voxel-level false discovery rate correction was used for the MDD dataset due to its reduced sample size. Finally, the binarized maps were overlaid to produce 2 network probability maps, which were thresholded at 50% to yield ketamine-induced hyper-functional and hypo-functional networks, respectively.

### Relation to canonical brain networks

For ease of interpretability, we investigated the spatial relations between the ketamine-induced brain functional alteration networks and 8 well-established canonical brain networks. The 7 cortical networks were defined as the visual, somatomotor, dorsal attention, ventral attention, limbic, frontoparietal, and default networks according to the Yeo et al. study [37]. The Human Brainnetome Atlas [38] was adopted to define the subcortical network including the amygdala, hippocampus, basal ganglia, and thalamus. The proportion of overlapping voxels between each ketamine-induced brain functional alteration network and a canonical network to all voxels within the corresponding canonical network was calculated to assess their spatial relation. Recently, a network correspondence toolbox (NCT) has been developed to provide a convenient means for computing Dice coefficients with spin test permutations to determine the magnitude and statistical significance of correspondence between user-defined maps and existing atlas labels [39]. Here, we used the NCT to test the statistical significance of the spatial correspondence between the ketamine-induced brain functional alteration networks and the canonical brain networks.

### Statistical significance testing

To determine whether the ketamine-induced brain functional alteration networks were statistically different from those generated by chance, we repeated the above-described FCNM procedure 1000 times with random contrast seeds, which yielded 1000 null networks. To quantify the similarity between the actual network and the 1000 null networks, we estimated their spatial overlap by calculating a Dice coefficient, defined as 2 × (overlapping voxels)/(network 1 voxels) + (network 2 voxels). A higher Dice coefficient indicates more similar networks.

### Validation analysis

Several validation analyses were performed to verify the robustness and consistency of our findings. To ensure that our results were independent of seed size, the FCNM procedure was repeated using 1- and 7-mm radius spheres. To examine the potential heterogeneity arising from different post-treatment scan timings, we conducted a sensitivity analysis by using the contrasts focusing on the acute phase of ketamine action (scanning within 48 hours post-administration). Finally, to investigate the potential influence of different imaging techniques, we conducted a validation analysis stratified by imaging modality.

## Results

### Included studies

Our analyses included a total of 16 studies with 20 within-subject pre- versus post-ketamine contrasts from 508 depressed patients with MDD or BD. Specifically, 12 studies with 16 contrasts from 406 MDD and 8 BD patients were included in the ketamine-induced hyper-functional network analysis. Eight studies with 8 contrasts from 145 MDD and 29 BD patients were included in the ketamine-induced hypo-functional network analysis. Our quality assessment identified 20 of the 24 contrasts as high quality, ensuring that our analysis relied on credible and robust data. Sample, treatment, and imaging information of the selected studies are provided in Tables S4 and S5 in the Supplementary Materials.

### Ketamine-induced brain functional alteration networks

**Figure 2.**
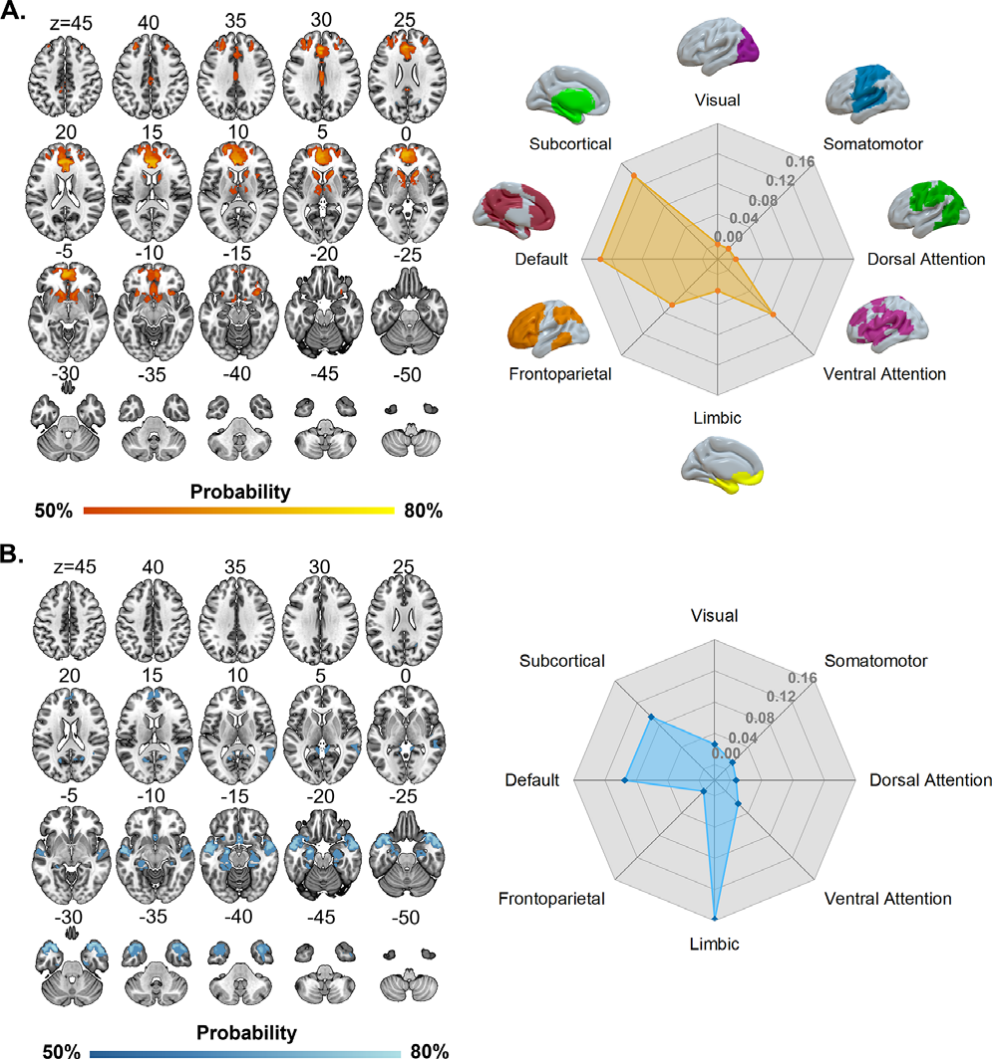
Ketamine-induced brain functional alteration networks derived from the HV dataset. (A) Ketamine-induced hyper-functional network (left panel) and its relation to canonical brain networks (right panel). (B) Ketamine-induced hypo-functional network (left panel) and its relation to canonical brain networks (right panel). Polar plots illustrate the proportion of overlapping voxels between each ketamine-induced brain functional alteration network and a canonical network to all voxels within the corresponding canonical network. Abbreviation: HV, healthy volunteer.

**Figure 3.**
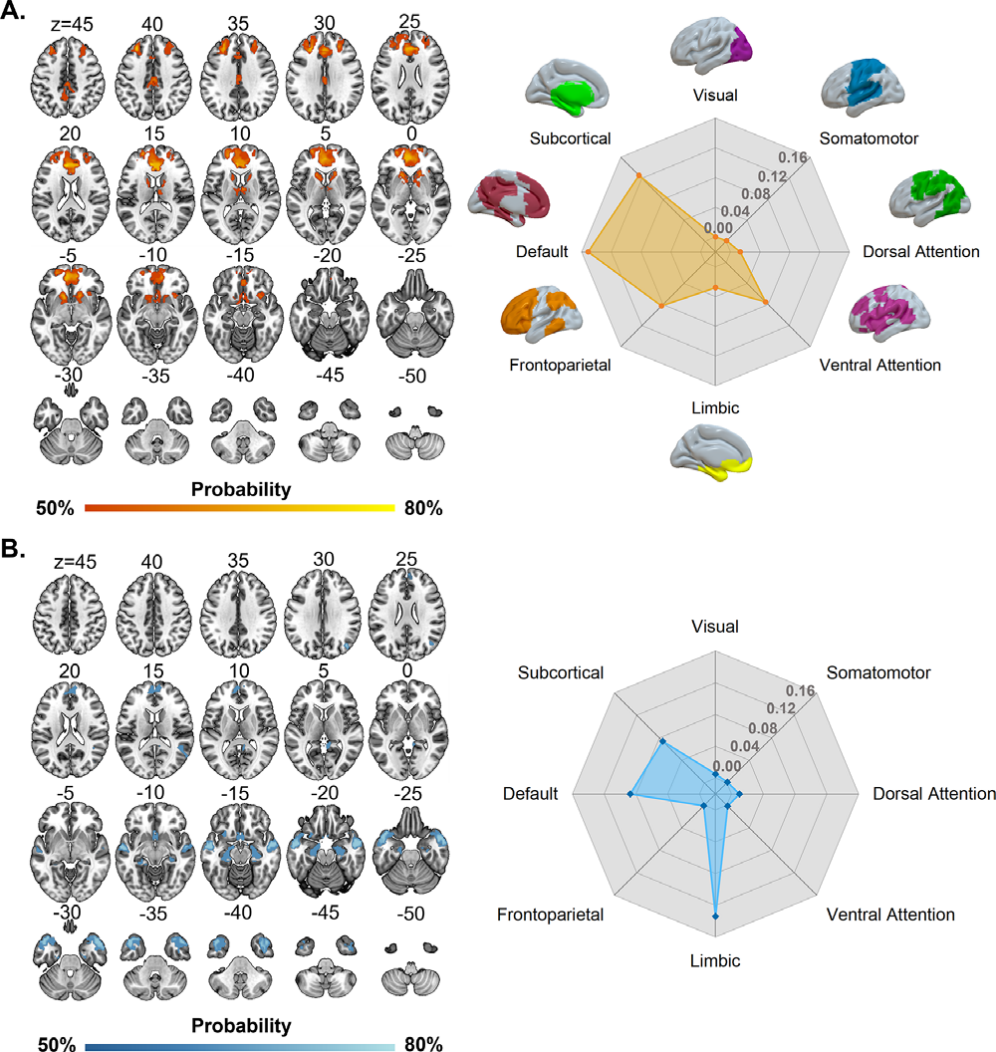
Ketamine-induced brain functional alteration networks derived from the MDD dataset. (A) Ketamine-induced hyper-functional network (left panel) and its relation to canonical brain networks (right panel). (B) Ketamine-induced hypo-functional network (left panel) and its relation to canonical brain networks (right panel). Polar plots illustrate the proportion of overlapping voxels between each ketamine-induced brain functional alteration network and a canonical network to all voxels within the corresponding canonical network. Abbreviation: MDD, major depressive disorder.

Analyses of both the HV and MDD datasets yielded similar ketamine-induced brain functional alteration networks, with subtle differences largely attributable to variation in sample sizes. The ketamine-induced hyper-functional network primarily included the bilateral caudate nucleus, thalamus, and medial prefrontal cortex (Figures 2A and 3A, left panel). With respect to canonical brain networks, the hyper-functional network principally implicated the subcortical (overlapping proportion: 13.67%) and default (overlapping proportion: 13.51%; Dice coefficient = 0.2882, *P*_NCT_ = 0.009) networks (Figures 2A and 3A, right panel and Table S6 in the Supplementary Materials). By contrast, the ketamine-induced hypo-functional network chiefly comprised the bilateral temporal pole, hippocampus, amygdala, and lateral temporal cortex (Figures 2B and 3B, left panel). As to canonical networks, the hypo-functional network predominantly implicated the limbic (overlapping proportion: 15.82%; Dice coefficient = 0.2757, *P*_NCT_ = 0.0779), subcortical (overlapping proportion: 9.45%), and default (overlapping proportion: 9.48%; Dice coefficient = 0.208, *P*_NCT_ = 0.0819) networks (Figures 2B and 3B, right panel and Table S6 in the Supplementary Materials).

### Statistical significance testing

By generating 1000 null networks with random contrast seeds and assessing their spatial overlap with the actual network, we observed low similarity of the actual ketamine-induced hyper-functional network (mean Dice coefficient ± SD: 0.050 ± 0.070) and hypo-functional network (0.065 ± 0.083) with their null counterparts (Figure S2 in the Supplementary Materials), suggesting that the ketamine-induced brain functional alteration networks were statistically different from those generated by chance.

### Validation analysis

Our comprehensive validation analyses confirmed the robustness of the main findings. First, regarding parameter selection, the FCNM procedure using 1-mm and 7-mm radius spheres yielded networks nearly identical to those using the 4-mm radius sphere (Figures S3 and S4 in the Supplementary Materials). Second, concerning the time window, the sensitivity analysis using the contrasts focusing on the acute phase of ketamine action (scanning within 48 hours post-administration) produced results consistent with the primary analysis (Figure S5 in the Supplementary Materials). Finally, regarding imaging modality, the ketamine-induced hyper-functional network derived from resting-state fMRI studies showed high spatial similarity to that in the main analysis (Figure S6 in the Supplementary Materials), suggesting that our findings were not driven by a single imaging modality. Note that the analyses for PET, cerebral blood flow (CBF), and task-based fMRI were not conducted due to the limited number of eligible studies.

## Discussion

By using a combination of the novel FCNM approach and large-scale brain functional connectome datasets, this is the first study to examine network localization of brain functional alterations induced by ketamine administration in depressed patients. Our analyses revealed 2 different and specific brain functional alteration networks related to ketamine treatment. The ketamine-induced hyper-functional network mainly involved the subcortical (caudate nucleus and thalamus) and default (medial prefrontal cortex) networks, while its hypo-functional counterpart predominantly implicated the limbic (temporal pole), subcortical (hippocampus and amygdala), and default (lateral temporal cortex) networks. These findings may not only help resolve previously reported discrepancies in ketamine-induced brain changes across studies, but also aid in elucidating the neurobiological effects of ketamine from a network perspective.

Considerable neuroimaging research effort in the past decade has been devoted to investigating the brain effects of ketamine treatment in depressed patients [10–14], yet inconsistency in the location and nature of the reported effects makes it difficult to unify this research. The substantial variability across studies may emerge for several reasons, including small sample sizes, heterogeneous patient populations, variable study designs, and different analytical strategies [40, 41]. In addition to these common challenges in neuroimaging, existing work has focused largely on regional brain effects, violating the concept that neural effects do not occur in isolation but rather rely on distributed, interconnected networks [15]. Moreover, convergent evidence suggests that several treatments for psychiatric populations show network-level brain representations and exert their salutary effects in a network-dependent manner [18–20, 42]. In this context, there is growing interest in the development and application of novel network-based approaches to localize psychiatric therapeutics to specific brain networks [31, 32]. In the current work, we adopted the FCNM approach to map the anatomically heterogeneous brain functional alterations following ketamine administration to common networks, highlighting the potential of this network-based framework to advance our understanding of the neurobiological effects of ketamine.

We found that the ketamine-induced hyper-functional network primarily included the caudate nucleus, thalamus, and medial prefrontal cortex, which is coherent with prior observations that depressed patients exhibit increased brain function in these regions after ketamine treatment [43–47]. Earlier brain imaging studies have shown lower brain function in the caudate nucleus, thalamus, and medial prefrontal cortex in depressed patients [48–52], indicating that the ketamine-induced increase in brain function may represent normalization or overcompensation. Although the exact mechanisms have been challenging to ascertain, there are several possible explanations [53]. Based on the disinhibition hypothesis, ketamine is proposed to selectively block NMDARs expressed on GABAergic inhibitory interneurons, which leads to a disinhibition of pyramidal neurons and enhanced glutamatergic firing. Additionally, ketamine can block NMDAR-mediated spontaneous neurotransmission, which results in the inhibition of the eukaryotic elongation factor 2 kinase activity and subsequently leads to an enhancement of brain-derived neurotrophic factor translation. Ketamine is also proposed to decrease excessive NMDAR-dependent burst firing activity of lateral habenula neurons to disinhibit downstream monoaminergic reward centers. These mechanisms of ketamine’s action are not mutually exclusive and may act in a complementary manner to account for the increased brain function.

Our data showed that the ketamine-induced hypo-functional network chiefly comprised the temporal pole, hippocampus, amygdala, and lateral temporal cortex. It is well established that the hippocampus and amygdala serve as pivotal hubs for emotion regulation and memory processing [54–56]. In depression, these regions often exhibit pathological hyperactivity, contributing to negative emotional bias and excessive memory retrieval [55, 57]. Therefore, the observed hypo-function may represent a critical normalization of the maladaptive hyperactivity typically seen in depression [57–60]. Regarding the lateral temporal cortex, its reduced activity might indicate a disruption of the default network. Since default network hyper-connectivity is linked to depressive rumination, ketamine may function by “uncoupling” the hyper-connectivity [61, 62]. From a circuit perspective, the subcortical inhibition might result from restored top-down control. The activated medial prefrontal cortex observed in our hyper-functional network could exert stronger inhibitory regulation over the limbic regions [63]. At the molecular level, ketamine may selectively block extrasynaptic GluN2B-containing NMDARs in the hippocampus. This blockade reduces tonic activation driven by ambient glutamate (background noise), thereby disinhibiting protein synthesis and enhancing synaptic plasticity mechanisms essential for antidepressant response [64, 65].

Our study is subject to several limitations. First, regarding temporal scope, most included studies focused on acute neural effects. The scarcity of extended follow-up data precludes us from fully characterizing the sustained, long-term impact of ketamine. Second, concerning methodological interpretation, FCNM relies on normative connectome data to generate network maps. Consequently, our results reflect the “network embedding” of ketamine-responsive sites within the healthy brain, rather than a direct quantification of drug-induced activation in every mapped region. Third, we acknowledge data heterogeneity. To maximize statistical power, we pooled coordinates from distinct imaging modalities (BOLD, PET, and CBF). While valid, these modalities capture different physiological processes, potentially introducing variance. Fourth, our analysis relied on published peak coordinates rather than raw neuroimaging data. This coordinate-based approach, while standard, inevitably lacks the spatial precision of mega-analyses based on original statistical maps. Finally, we focused exclusively on functional metrics and employed a consensus threshold. While excluding structural data and setting a 50% overlap ensures specific and robust findings, these choices may omit subtle or trait-related structural alterations.

In conclusion, the current study opens new perspectives by being the first to investigate network localization of ketamine-induced brain functional alterations in depressed patients. By combining the novel FCNM approach and large-scale brain functional connectome datasets, we mapped the anatomically heterogeneous brain functional alterations (increase and decrease) associated with ketamine treatment to specific hyper-functional and hypo-functional networks. These findings may shed light on the neurobiological effects of ketamine from a network perspective, which might represent a crucial step toward fostering the clinical application of ketamine in antidepressant treatment.

## Supporting information

10.1192/j.eurpsy.2026.10164.sm001 Ma et al. supplementary material Ma et al. supplementary material

## Data Availability

The data and analysis codes used in the preparation of this article are publicly available at https://osf.io/w2mp5/.

## Supplementary material

The supplementary material for this article can be found at http://doi.org/10.1192/j.eurpsy.2026.10164.

## References

[1] Malhi GS, Mann JJ. Depression. Lancet. 2018;392:2299–312. 30396512 10.1016/S0140-6736(18)31948-2

[2] Otte C, Gold SM, Penninx BW, Pariante CM, Etkin A, Fava M, et al. Major depressive disorder. Nat Rev Dis Primers. 2016;2:16065. 27629598 10.1038/nrdp.2016.65

[3] Rush AJ, Thase ME. Improving depression outcome by patient-Centered medical management. Am J Psychiatry. 2018;175:1187–98. 30220219 10.1176/appi.ajp.2018.18040398

[4] Krystal JH, Abdallah CG, Sanacora G, Charney DS, Duman RS. Ketamine: a paradigm shift for depression research and treatment. Neuron. 2019;101:774–8. 30844397 10.1016/j.neuron.2019.02.005 PMC6560624

[5] McIntyre RS, Rosenblat JD, Nemeroff CB, Sanacora G, Murrough JW, Berk M, et al. Synthesizing the evidence for ketamine and Esketamine in treatment-resistant depression: an international expert opinion on the available evidence and implementation. Am J Psychiatry. 2021;178:383–99. 33726522 10.1176/appi.ajp.2020.20081251 PMC9635017

[6] Coyle CM, Laws KR. The use of ketamine as an antidepressant: a systematic review and meta-analysis. Hum Psychopharmacol. 2015;30:152–63. 25847818 10.1002/hup.2475

[7] Yang Y, Cui Y, Sang K, Dong Y, Ni Z, Ma S, et al. Ketamine blocks bursting in the lateral habenula to rapidly relieve depression. Nature. 2018;554:317–22. 29446381 10.1038/nature25509

[8] Cui Y, Hu S, Hu H. Lateral Habenular burst firing as a target of the rapid antidepressant effects of ketamine. Trends Neurosci. 2019;42:179–91. 30823984 10.1016/j.tins.2018.12.002

[9] Ma S, Chen M, Jiang Y, Xiang X, Wang S, Wu Z, et al. Sustained antidepressant effect of ketamine through NMDAR trapping in the LHb. Nature. 2023;622:802–9. 37853123 10.1038/s41586-023-06624-1 PMC10600008

[10] Zavaliangos-Petropulu A, Al-Sharif NB, Taraku B, Leaver AM, Sahib AK, Espinoza RT, et al. Neuroimaging-derived biomarkers of the antidepressant effects of ketamine. Biol Psychiatry Cogn Neurosci Neuroimaging. 2023;8:361–86. 36775711 10.1016/j.bpsc.2022.11.005 PMC11483103

[11] Alario AA, Niciu MJ. Biomarkers of ketamine’s antidepressant effect: a clinical review of genetics, functional connectivity, and neurophysiology. Chronic Stress (Thousand Oaks). 2021;5:24705470211014210. 34159281 10.1177/24705470211014210 PMC8186113

[12] Medeiros GC, Matheson M, Demo I, Reid MJ, Matheson S, Twose C, et al. Brain-based correlates of antidepressant response to ketamine: a comprehensive systematic review of neuroimaging studies. Lancet Psychiatry. 2023;10:790–800. 37625426 10.1016/S2215-0366(23)00183-9 PMC11534374

[13] Ionescu DF, Felicione JM, Gosai A, Cusin C, Shin P, Shapero BG, et al. Ketamine-associated brain changes: a review of the neuroimaging literature. Harv Rev Psychiatry. 2018;26:320–39. 29465479 10.1097/HRP.0000000000000179 PMC6102096

[14] Kotoula V, Webster T, Stone J, Mehta MA. Resting-state connectivity studies as a marker of the acute and delayed effects of subanaesthetic ketamine administration in healthy and depressed individuals: a systematic review. Brain Neurosci Adv. 2021;5:23982128211055426. 34805548 10.1177/23982128211055426 PMC8597064

[15] Fornito A, Zalesky A, Breakspear M. The connectomics of brain disorders. Nat Rev Neurosci. 2015;16:159–72. 25697159 10.1038/nrn3901

[16] Taylor JJ, Siddiqi SH, Fox MD. Coordinate network mapping: an emerging approach for morphometric meta-analysis. Am J Psychiatry. 2021;178:1080–1. 34855450 10.1176/appi.ajp.2021.21100987 PMC12224197

[17] Fox MD. Mapping symptoms to brain networks with the human connectome. N Engl J Med. 2018;379:2237–45. 30575457 10.1056/NEJMra1706158

[18] Sobesky L, Goede L, Odekerken VJJ, Wang Q, Li N, Neudorfer C, et al. Subthalamic and pallidal deep brain stimulation: are we modulating the same network? Brain. 2022;145:251–62. 34453827 10.1093/brain/awab258

[19] Siddiqi SH, Schaper F, Horn A, Hsu J, Padmanabhan JL, Brodtmann A, et al. Brain stimulation and brain lesions converge on common causal circuits in neuropsychiatric disease. Nat Hum Behav. 2021;5:1707–16. 34239076 10.1038/s41562-021-01161-1 PMC8688172

[20] Scangos KW, State MW, Miller AH, Baker JT, Williams LM. New and emerging approaches to treat psychiatric disorders. Nat Med. 2023;29:317–33. 36797480 10.1038/s41591-022-02197-0 PMC11219030

[21] Cheng Y, Cai H, Liu S, Yang Y, Pan S, Zhang Y, et al. Brain network localization of Gray matter atrophy and neurocognitive and social cognitive dysfunction in schizophrenia. Biol Psychiatry. 2025;97:148–56. 39103010 10.1016/j.biopsych.2024.07.021

[22] Zhang X, Xu R, Ma H, Qian Y, Zhu J. Brain structural and functional damage network localization of suicide. Biol Psychiatry. 2024;95:1091–9. 38215816 10.1016/j.biopsych.2024.01.003

[23] Xu R, Zhang X, Zhou S, Guo L, Mo F, Ma H, et al. Brain structural damage networks at different stages of schizophrenia. Psychol Med. 2024;1–11. doi:10.1017/S0033291724003088. 39660416

[24] Mo F, Zhao H, Li Y, Cai H, Song Y, Wang R, et al. Network localization of State and trait of auditory verbal hallucinations in schizophrenia. Schizophr Bull. 2024;50:1326–36. 38401526 10.1093/schbul/sbae020 PMC11548935

[25] Padmanabhan JL, Cooke D, Joutsa J, Siddiqi SH, Ferguson M, Darby RR, et al. A human depression circuit derived from focal brain lesions. Biol Psychiatry. 2019;86:749–58. 31561861 10.1016/j.biopsych.2019.07.023 PMC7531583

[26] Trapp NT, Bruss JE, Manzel K, Grafman J, Tranel D, Boes AD. Large-scale lesion symptom mapping of depression identifies brain regions for risk and resilience. Brain. 2023;146:1672–85. 36181425 10.1093/brain/awac361 PMC10319784

[27] Zhukovsky P, Anderson JAE, Coughlan G, Mulsant BH, Cipriani A, Voineskos AN. Coordinate-based network mapping of brain structure in major depressive disorder in younger and older adults: a systematic review and meta-analysis. Am J Psychiatry. 2021;178:1119–28. 34645274 10.1176/appi.ajp.2021.21010088

[28] Taylor JJ, Lin C, Talmasov D, Ferguson MA, Schaper F, Jiang J, et al. A transdiagnostic network for psychiatric illness derived from atrophy and lesions. Nat Hum Behav. 2023;7:420–9. 36635585 10.1038/s41562-022-01501-9 PMC10236501

[29] Darby RR, Joutsa J, Fox MD. Network localization of heterogeneous neuroimaging findings. Brain. 2019;142:70–9. 30551186 10.1093/brain/awy292 PMC6308311

[30] Jones BDM, Zhukovsky P, Hawco C, Ortiz A, Cipriani A, Voineskos AN, et al. Protocol for a systematic review and meta-analysis of coordinate-based network mapping of brain structure in bipolar disorder across the lifespan. BJPsych Open. 2023;9:e178. 37811544 10.1192/bjo.2023.569 PMC10594157

[31] Fox MD, Buckner RL, Liu H, Chakravarty MM, Lozano AM, Pascual-Leone A. Resting-state networks link invasive and noninvasive brain stimulation across diverse psychiatric and neurological diseases. Proc Natl Acad Sci USA. 2014;111:E4367–75. 25267639 10.1073/pnas.1405003111 PMC4205651

[32] Siddiqi SH, Taylor SF, Cooke D, Pascual-Leone A, George MS, Fox MD. Distinct symptom-specific treatment targets for circuit-based neuromodulation. Am J Psychiatry. 2020;177:435–46. 32160765 10.1176/appi.ajp.2019.19090915 PMC8396109

[33] Yan CG, Wang XD, Zuo XN, Zang YF. DPABI: Data Processing & Analysis for (resting-state) brain imaging. Neuroinformatics. 2016;14:339–51. 27075850 10.1007/s12021-016-9299-4

[34] Ashburner J. A fast diffeomorphic image registration algorithm. NeuroImage. 2007;38:95–113. 17761438 10.1016/j.neuroimage.2007.07.007

[35] Murphy K, Birn RM, Handwerker DA, Jones TB, Bandettini PA. The impact of global signal regression on resting state correlations: are anti-correlated networks introduced? NeuroImage. 2009;44:893–905. 18976716 10.1016/j.neuroimage.2008.09.036 PMC2750906

[36] Murphy K, Fox MD. Towards a consensus regarding global signal regression for resting state functional connectivity MRI. NeuroImage. 2017;154:69–73. 10.1016/j.neuroimage.2016.11.052 PMC5489207 27888059

[37] Yeo BT, Krienen FM, Sepulcre J, Sabuncu MR, Lashkari D, Hollinshead M, et al. The organization of the human cerebral cortex estimated by intrinsic functional connectivity. J Neurophysiol. 2011;106:1125–65. 21653723 10.1152/jn.00338.2011 PMC3174820

[38] Fan L, Li H, Zhuo J, Zhang Y, Wang J, Chen L, et al. The human Brainnetome atlas: a new brain atlas based on connectional architecture. Cereb Cortex. 2016;26:3508–26. 27230218 10.1093/cercor/bhw157 PMC4961028

[39] Kong R, Spreng RN, Xue A, Betzel RF, Cohen JR, Damoiseaux JS, et al. A network correspondence toolbox for quantitative evaluation of novel neuroimaging results. Nat Commun. 2025;16:2930. 40133295 10.1038/s41467-025-58176-9 PMC11937327

[40] Poldrack RA, Baker CI, Durnez J, Gorgolewski KJ, Matthews PM, Munafo MR, et al. Scanning the horizon: towards transparent and reproducible neuroimaging research. Nat Rev Neurosci. 2017;18:115–26. 28053326 10.1038/nrn.2016.167 PMC6910649

[41] Etkin A. A reckoning and research agenda for neuroimaging in psychiatry. Am J Psychiatry. 2019;176:507–11. 31256624 10.1176/appi.ajp.2019.19050521

[42] Horn A, Reich M, Vorwerk J, Li N, Wenzel G, Fang Q, et al. Connectivity predicts deep brain stimulation outcome in Parkinson disease. Ann Neurol. 2017;82:67–78. 28586141 10.1002/ana.24974 PMC5880678

[43] Gonzalez S, Vasavada M, Njau S, Sahib AK, Espinoza R, Narr KL, et al. Acute changes in cerebral blood flow after single-infusion ketamine in major depression: a pilot study. Neurol Psychiatry Brain Res. 2020;38:5–11. 34887623 10.1016/j.npbr.2020.08.006 PMC8653983

[44] Mkrtchian A, Evans JW, Kraus C, Yuan P, Kadriu B, Nugent AC, et al. Ketamine modulates fronto-striatal circuitry in depressed and healthy individuals. Mol Psychiatry. 2021;26:3292–301. 32929215 10.1038/s41380-020-00878-1 PMC8462973

[45] Abdallah CG, Averill LA, Collins KA, Geha P, Schwartz J, Averill C, et al. Ketamine treatment and global brain connectivity in major depression. Neuropsychopharmacology. 2017;42:1210–9. 27604566 10.1038/npp.2016.186 PMC5437875

[46] Abdallah CG, Dutta A, Averill CL, McKie S, Akiki TJ, Averill LA, et al. Ketamine, but not the NMDAR antagonist Lanicemine, increases prefrontal global connectivity in depressed patients. Chronic Stress (Thousand Oaks). 2018;2. 10.1177/2470547018796102 PMC6154502 30263977

[47] Hung CC, Zhang S, Chen CM, Duann JR, Lin CP, Lee TS, et al. Striatal functional connectivity in chronic ketamine users: a pilot study. Am J Drug Alcohol Abuse. 2020;46:31–43. 31264888 10.1080/00952990.2019.1624764 PMC8627683

[48] Wu QZ, Li DM, Kuang WH, Zhang TJ, Lui S, Huang XQ, et al. Abnormal regional spontaneous neural activity in treatment-refractory depression revealed by resting-state fMRI. Hum Brain Mapp. 2011;32:1290–9. 20665717 10.1002/hbm.21108 PMC6870367

[49] Strakowski SM, Delbello MP, Adler CM. The functional neuroanatomy of bipolar disorder: a review of neuroimaging findings. Mol Psychiatry. 2005;10:105–16. 15340357 10.1038/sj.mp.4001585

[50] Pizzagalli DA, Holmes AJ, Dillon DG, Goetz EL, Birk JL, Bogdan R, et al. Reduced caudate and nucleus accumbens response to rewards in unmedicated individuals with major depressive disorder. Am J Psychiatry. 2009;166:702–10. 19411368 10.1176/appi.ajp.2008.08081201 PMC2735451

[51] Ma C, Ding J, Li J, Guo W, Long Z, Liu F, et al. Resting-state functional connectivity bias of middle temporal gyrus and caudate with altered gray matter volume in major depression. PLoS One. 2012;7:e45263. 23028892 10.1371/journal.pone.0045263 PMC3454420

[52] Lui S, Wu Q, Qiu L, Yang X, Kuang W, Chan RC, et al. Resting-state functional connectivity in treatment-resistant depression. Am J Psychiatry. 2011;168:642–8. 21362744 10.1176/appi.ajp.2010.10101419

[53] Zanos P, Gould TD. Mechanisms of ketamine action as an antidepressant. Mol Psychiatry. 2018;23:801–11. 29532791 10.1038/mp.2017.255 PMC5999402

[54] Phelps EA. Human emotion and memory: interactions of the amygdala and hippocampal complex. Curr Opin Neurobiol. 2004;14:198–202. 15082325 10.1016/j.conb.2004.03.015

[55] Disner SG, Beevers CG, Haigh EA, Beck AT. Neural mechanisms of the cognitive model of depression. Nat Rev Neurosci. 2011;12:467–77. 21731066 10.1038/nrn3027

[56] Phelps EA, LeDoux JE. Contributions of the amygdala to emotion processing: from animal models to human behavior. Neuron. 2005;48:175–87. 16242399 10.1016/j.neuron.2005.09.025

[57] Siegle GJ, Carter CS, Thase ME. Use of FMRI to predict recovery from unipolar depression with cognitive behavior therapy. Am J Psychiatry. 2006;163:735–8. 16585452 10.1176/ajp.2006.163.4.735

[58] Scheidegger M, Henning A, Walter M, Lehmann M, Kraehenmann R, Boeker H, et al. Ketamine administration reduces amygdalo-hippocampal reactivity to emotional stimulation. Hum Brain Mapp. 2016;37:1941–52. 26915535 10.1002/hbm.23148 PMC6867525

[59] Sheline YI, Barch DM, Donnelly JM, Ollinger JM, Snyder AZ, Mintun MA. Increased amygdala response to masked emotional faces in depressed subjects resolves with antidepressant treatment: an fMRI study. Biol Psychiatry. 2001;50:651–8. 11704071 10.1016/s0006-3223(01)01263-x

[60] Drevets WC, Bogers W, Raichle ME. Functional anatomical correlates of antidepressant drug treatment assessed using PET measures of regional glucose metabolism. Eur Neuropsychopharmacol. 2002;12:527–44. 12468016 10.1016/s0924-977x(02)00102-5

[61] Scheidegger M, Walter M, Lehmann M, Metzger C, Grimm S, Boeker H, et al. Ketamine decreases resting state functional network connectivity in healthy subjects: implications for antidepressant drug action. PLoS One. 2012;7:e44799. 23049758 10.1371/journal.pone.0044799 PMC3461985

[62] Evans JW, Szczepanik J, Brutsché N, Park LT, Nugent AC, Zarate CA. Default mode connectivity in major depressive disorder measured up to 10 days after ketamine administration. Biol Psychiatry. 2018;84:582–90. 29580569 10.1016/j.biopsych.2018.01.027 PMC6093808

[63] Hare BD, Duman RS. Prefrontal cortex circuits in depression and anxiety: contribution of discrete neuronal populations and target regions. Mol Psychiatry. 2020;25:2742–58. 32086434 10.1038/s41380-020-0685-9 PMC7442605

[64] Miller OH, Yang L, Wang CC, Hargroder EA, Zhang Y, Delpire E, et al. GluN2B-containing NMDA receptors regulate depression-like behavior and are critical for the rapid antidepressant actions of ketamine. elife. 2014;3:e03581. 25340958 10.7554/eLife.03581 PMC4270067

[65] Autry AE, Adachi M, Nosyreva E, Na ES, Los MF, Cheng PF, et al. NMDA receptor blockade at rest triggers rapid behavioural antidepressant responses. Nature. 2011;475:91–5. 21677641 10.1038/nature10130 PMC3172695

