# Supplementary material for: Network localization of brain functional effects of ketamine treatment for major depression
Source: Eur Psychiatry. 2026 Feb 13;69(1):e28. doi: 10.1192/j.eurpsy.2026.10164 (PMC12978991; doi:10.1192/j.eurpsy.2026.10164)
Supplement: Ma et al. supplementary material [file S0924933826101643sup001.docx]

**Supplementary materials**

**Table S1. Detailed scoring system and criteria used for the quality assessment of the included studies**

| Criteria | Items | 2 | 1 | 0 |
| --- | --- | --- | --- | --- |
| Participants & Selection | Item 1: Sample Size & Statistical Power | Adequate Power: N ≥ 20 per group (or Total N ≥ 40 for moderate effect sizes) | 10 ≤ N < 20 per group | N < 10 per group (Statistical power critically low) |
|  | Item 2: Diagnosis & Washout Status | Strict Standards: Diagnosis via structured interview (e.g., SCID/MINI) and clearly defined washout period (drug-free or strict stability) | Clinical/Lenient: Diagnosis by clinician impression only or washout reported but allowed concurrent medication changes | Unclear: Diagnostic criteria not specified or medication status confounding |
| Study Design & Treatment Protocol | Item 3: Design & Control | Gold Standard: RCT with Placebo/Active Control and double-blind design | Controlled but Limited: Non-randomized controlled trial or single-blind design | Weak Design: Open-label (Pre-post only) with no control group or Retrospective design |
|  | Item 4: Ketamine Protocol | Standardized: IV Infusion (0.5 mg/kg over 40 min) or validated equivalent | Bolus/Variant: 0.5 mg/kg  in a bolus injection (< 5 min) or other non-standard duration | Unreported: IV infusion (0.5 mg/kg) but infusion duration is not reported |
|  | Item 5: Scan Timing | Scan timing strictly matches PK/PD windows (e.g., 4h, 24h post) | Scan timing window is broad | Scan timing vague/disconnected from drug effects |
| Imaging Quality (Modality Agnostic) | Item 6: Acquisition & Motion Control | High Quality: High field strength (3T+ MRI) or High-Res PET/CT; and rigorous motion correction (e.g., FD scrubbing for fMRI; Frame realignment for PET) | Acceptable: 1.5T MRI or Older PET; or standard motion correction (parameters regress) without aggressive exclusion | Low Quality: Parameters missing or no mention of motion control |
|  | Item 7: Signal Quantification & Preprocessing | Validated Pipeline: Standard software (SPM/FSL) and gold-standard quantification (e.g., Kinetic Modeling/VT​ for PET; Validated Seed/ICA for fMRI) | Standard: Standard preprocessing but semi-quantitative metrics (e.g., SUV/SUVr for PET; ALFF/ReHo without physiological correction) | Flawed/Unclear: Custom code without validation or reference region/baseline clearly inappropriate |
| Statistical Rigor | Item 8: Multiple Comparison Correction | Strict Correction: Voxel/Cluster-level correction (FWE, FDR, or TFCE) at p < 0.05 | Liberal/Exploratory: Uncorrected p < 0.001 with cluster threshold (k > 10) | Invalid: Uncorrected p < 0.05 or ROI picked post-hoc |
|  | Item 9: Longitudinal Modeling | Interaction Model: Uses LMM (Linear Mixed Models) or Repeated Measures ANOVA to test Group × Time interaction | Simple Changes: Reports only Paired t-tests (Pre vs. Post) without direct comparison to control group change | Inappropriate: Statistical method not described or incorrect for longitudinal data |

Abbreviations: ALFF, amplitude of low frequency fluctuations; ANOVA, analysis of variance; CBF, cerebral blood flow; FD, framewise displacement; FDR, false discovery rate; fMRI, functional magnetic resonance imaging; FWE, family-wise error; ICA, independent component analysis; IV, intravenous; LMM, linear mixed models; MINI, Mini-International Neuropsychiatric Interview; MRI, magnetic resonance imaging; PET, positron emission tomography; PK/PD, Pharmacokinetics/Pharmacodynamics; RCT, randomized controlled trial; ReHo, regional homogeneity; ROI, region of interest; SCID, Structured Clinical Interview for DSM; SD, standard deviation; SUV, standardized uptake value; SUVr, standardized uptake value ratio; TFCE, threshold-free cluster enhancement; VT, total distribution volume.

**Table S2. Quality assessment results for each included study**

| Study | Participants & Selection | | Study Design & Treatment Protocol | | | | Imaging Quality (Modality Agnostic) | | Statistical Rigor | | Total Score |
| --- | --- | --- | --- | --- | --- | --- | --- | --- | --- | --- | --- |
|  | Item 1: Sample Size & Statistical Power | Item 2: Diagnosis & Washout Status | | Item 3: Design & Control | Item 4: Ketamine Protocol | Item 5: Scan Timing | Item 6: Acquisition & Motion Control | Item 7: Signal Quantification & Preprocessing | Item 8: Multiple Comparison Correction | Item 9: Longitudinal Modeling |  |
| Carlson et al., 2013 [1] | 2 | 2 | 0 | | 2 | 2 | 2 | 2 | 2 | 1 | 15 |
| Murrough et al., 2015-1 [2] | 1 | 2 | 1 | | 2 | 2 | 2 | 2 | 2 | 1 | 15 |
| Murrough et al., 2015-2 [2] | 1 | 2 | 1 | | 2 | 2 | 2 | 2 | 2 | 1 | 15 |
| Li et al., 2016 [3] | 2 | 2 | 2 | | 2 | 2 | 1 | 1 | 2 | 2 | 16 |
| Abdallah et al., 2017a [4] | 2 | 2 | 1 | | 2 | 2 | 1 | 2 | 1 | 1 | 14 |
| Abdallah et al., 2017b [5] | 2 | 2 | 2 | | 2 | 2 | 2 | 2 | 2 | 2 | 18 |
| Reed et al., 2018 [6] | 2 | 2 | 2 | | 2 | 2 | 2 | 1 | 2 | 2 | 17 |
| Sterpenich et al., 2019-1 [7] | 1 | 2 | 0 | | 1 | 2 | 1 | 2 | 2 | 2 | 13 |
| Sterpenich et al., 2019-2 [7] | 1 | 2 | 0 | | 1 | 2 | 1 | 2 | 2 | 2 | 13 |
| Gonzalez et al., 2020 [8] | 1 | 2 | 0 | | 2 | 2 | 2 | 2 | 2 | 2 | 15 |
| Rivas-Grajales et al., 2021 [9] | 2 | 2 | 0 | | 2 | 2 | 2 | 2 | 2 | 1 | 15 |
| Liu et al., 2023 [10] | 2 | 2 | 0 | | 2 | 1 | 2 | 2 | 2 | 1 | 14 |
| Rengasamy et al., 2024-1 [11] | 2 | 1 | 2 | | 0 | 2 | 2 | 2 | 2 | 2 | 15 |
| Rengasamy et al., 2024-2 [11] | 2 | 1 | 2 | | 0 | 2 | 2 | 2 | 2 | 2 | 15 |
| Chen et al., 2023-1 [12] | 2 | 2 | 0 | | 2 | 1 | 2 | 2 | 2 | 1 | 14 |
| Chen et al., 2023-2 [12] | 2 | 2 | 0 | | 2 | 1 | 2 | 2 | 2 | 1 | 14 |
| Carlson et al., 2013 [1] | 2 | 2 | 0 | | 2 | 2 | 2 | 2 | 2 | 1 | 15 |
| Nugent et al., 2014 [13] | 2 | 2 | 2 | | 0 | 2 | 1 | 2 | 2 | 1 | 14 |
| Li et al., 2016 [3] | 2 | 2 | 2 | | 2 | 2 | 1 | 1 | 2 | 2 | 16 |
| Reed et al., 2018 [6] | 2 | 2 | 2 | | 2 | 2 | 2 | 1 | 2 | 2 | 17 |
| Chen et al., 2019 [14] | 2 | 2 | 2 | | 2 | 2 | 1 | 2 | 2 | 1 | 16 |
| Gonzalez et al., 2020 [8] | 1 | 2 | 0 | | 2 | 2 | 2 | 2 | 2 | 2 | 15 |
| Zhang et al., 2023 [15] | 1 | 2 | 0 | | 2 | 1 | 2 | 2 | 2 | 1 | 13 |
| Wang et al., 2022 [16] | 2 | 1 | 0 | | 2 | 1 | 2 | 2 | 2 | 1 | 13 |

Studies were evaluated based on four domains: Participants & Selection, Study Design & Treatment Protocol, Imaging Quality, and Statistical Rigor. The total score ranges from 0 to 18. High Quality: 14–18 points; Moderate Quality: 9–13 points; Low Quality: 0–8 points.

**Table S3. Demographic and clinical characteristics of the HV and MDD datasets**

| **Characteristic** | **HV dataset** | **MDD dataset** |
| --- | --- | --- |
| Sample size | 1113 | 255 |
| Age (years) | 32.66 ± 12.78 | 40.99 ± 12.67 |
| Gender (F/M) | 643/470 | 161/94 |
| Handedness (right/left/both) | 1110/3/0 | 252/3/0 |
| Education (years) | - | 10.48 ± 3.93 |
| First episode/recurrence | - | 95/160 |
| Onset age (years) | - | 35.47 ± 12.24 |
| Duration of illness (years) | - | 2.50 (0.58, 7.00) |
| HAMA | - | 19.60 ± 7.61 |
| HAMD | - | 30.68 ± 10.81 |
| FD (mm) | 0.13 ± 0.06 | 0.13 ± 0.09 |

Data are expressed as mean ± standard deviation or median (Q1, Q3). Abbreviations: F, female; FD, frame-wise displacement; HAMA, the 14-item Hamilton Rating Scale for Anxiety; HAMD, the 24-item Hamilton Rating Scale for Depression; HV, healthy volunteer; M, male; MDD, major depressive disorder; Q, quartiles.

**Table S4. Sample, treatment, and imaging information of the studies included in the ketamine-induced hyper-functional network analysis**

| **Study** | **Diagnosis** | **Patients**  **N/F** | **Age, years**  **Mean ± SD/**  **Mean (quartiles)** | **Clinical assessment** | | | **Neuroimaging time points** | **Design** | **Application** | **Neuroimaging measure** |
| --- | --- | --- | --- | --- | --- | --- | --- | --- | --- | --- |
|  |  |  |  | **Instrument** | **Pre-ketamine**  **Mean ± SD** | **Post-ketamine**  **Mean ± SD** |  |  |  |  |
| Carlson et al., 2013 [1] | MDD  (DSM-IV) | 20/6 | 47.6 ± 12.2 | MADRS | 33.1 ± 5.7 | 18.7 ± 8.2 (40 min) | Baseline, 120 min | Open label | 0.5 mg/kg over 40 min | CMRGlu |
| Murrough et al., 2015-1 [2] | MDD  (DSM-IV) | 18/8 | 38.1 ± 13.8 | MADRS | 29.9 ± 6.8 | 16.4 ± 11.1 | Baseline, 1 d | RCT | 0.5 mg/kg over 40 min | Task-induced activation |
| Murrough et al., 2015-2 [2] | MDD  (DSM-IV) | 18/8 | 38.1 ± 13.8 | MADRS | 29.9 ± 6.8 | 16.4 ± 11.1 | Baseline, 1 d | RCT | 0.5 mg/kg over 40 min | Task-induced activation |
| Li et al., 2016 [3] | MDD  (DSM-IV) | 16/11 | 43.3 ± 11.9 | HDRS-17 | 22.6 ± 5.8 | -32.8% ± 27.2%  (40 min) | Baseline, 40 min | RCT | 0.5 mg/kg over 40 min | SUV of glucose metabolism |
| Abdallah et al., 2017a [4] | MDD  (DSM-IV) | 18/8 | 43 ± 2.2 | MADRS | 29.4 ± 1.6 | 15.2 ± 3.5 | Baseline, 1 d | Open label | 0.5 mg/kg over 40 min | GBCr |
| Abdallah et al., 2017b [5] | MDD  (DSM-IV) | 22/10 | 44.8 ± 2.3 | MADRS | 32 ± 1.2 | NA | Baseline, 1 d | RCT | 0.5 mg/kg over 40 min | GBCr |
| Reed et al., 2018 [6] | MDD  (DSM-IV) | 33/21 | 36.06 ± 9.74 | MADRS | 33.96 ± 4.64 | 24.78 ± 10.04 | Baseline, 2 d | RCT | 0.5 mg/kg over 40 min | Task-induced activation |
| Sterpenich et al., 2019-1 [7] | MDD  (DSM-IV) | 10/6 | 51 (38, 58) | MADRS | ≥ 25 | NA | Baseline, 1 d, 7 d | Open label | 0.5 mg/kg  in a bolus injection over 1 min | Task-induced activation |
| Sterpenich et al., 2019-2 [7] | MDD  (DSM-IV) | 10/6 | 51 (38, 58) | MADRS | ≥ 25 | NA | Baseline, 1 d, 7 d | Open label | 0.5 mg/kg  in a bolus injection over 1 min | Task-induced activation |
| Gonzalez et al., 2020 [8] | MDD  (DSM-IV) | 11/3 | 47.7 ± 11.9 | HDRS-17 | 22.44 ± 3.68 | 9.00 ± 7.67 (1 d) | Baseline, 1 h, 6 h, 1 d | Open label | 0.5 mg/kg over 40 min | CBF |
|  |  |  |  | MADRS | 34.89 ± 3.44 | 16.00 ± 13.58 (1 d) |  |  |  |  |
|  |  |  |  | QIDS-SR | 17.78 ± 7.60 | 10.40 ± 6.69 (1 d) |  |  |  |  |
| Rivas-Grajales et al., 2021 [9] | MDD  (DSM-IV) | 35/16 | 42.2 ± 13.9 | MADRS | 30.6 ± 5.2 | 13.7 ± 9.4 | Baseline, 1 d | RCT | 0.5 mg/kg over 40 min | FC |
|  |  |  |  | QIDS-SR | 16.2 ± 4.4 | 8.5 ± 6.4 |  |  |  |  |
| Liu et al., 2023 [10] | MDD  (DSM-V) | 39/24 | 36.5 ± 12.1 | HAMD-17 | 23.2 ± 4.6 | 12.2 ± 7.4 | Baseline, 24 h (after 6th infusion, 2 w) | Open label | 0.5 mg/kg over 40 min | FC |
|  |  |  |  | SSI | 9.0 ± 3.5 | 6.0 ± 2.3 |  |  |  |  |
| Rengasamy et al., 2024-1 [11] | MDD | 152/95 | 34.2 ± 10.6 | MADRS | 32.6 ± 5.2 | -41.6% ± 29.4% | Baseline, 1 d | RCT | 0.5 mg/kg | FC |
| Rengasamy et al., 2024-2 [11] | MDD | 152/95 | 34.2 ± 10.6 | MADRS | 32.6 ± 5.2 | -41.6% ± 29.4% | Baseline, 1 d | RCT | 0.5 mg/kg | FC |
| Chen et al., 2023-1 [12] | 22MDD 3BD  (DSM-V) | 25/13 | 37.20 ± 11.36 | HAMD-17 | 23.28 ± 3.89 | NA | Baseline, 24 h (after 6th infusion, 12 d) | Open label | 0.5 mg/kg | FC |
|  |  |  |  | SSI | 4.72 ± 2.32 | NA |  |  |  |  |
| Chen et al., 2023-2 [12] | 10MDD 5BD  (DSM-V) | 15/8 | 25.60 ± 6.94 | HAMD-17 | 25.20 ± 5.67 | NA | Baseline, 24 h (after 6th infusion, 12 d) | Open label | 0.5 mg/kg | FC |
|  |  |  |  | SSI | 6.87 ± 2.80 | NA |  |  |  |  |

Abbreviations: BD, bipolar disorder; CBF: cerebral blood flow; CMRGlu, cerebral metabolic rate for glucose; DSM-IV, Diagnostic and Statistical Manual of Mental Disorders, fourth edition; DSM-V, Diagnostic and Statistical Manual of Mental Disorders, fifth edition; F, female; FC, functional connectivity; GBCr, global brain connectivity with global signal regression; HAMD-17/HDRS-17, Hamilton Depression Rating Scale, 17 item; MADRS, Montgomery Asberg Depression Rating Scale; MDD, major depressive disorder; N, number; NA, not available; QIDS-SR, Quick Inventory of Depressive Symptoms, self-report; RCT, randomized controlled trial; rMRGlu, regional metabolic rate of glucose; SSI, Beck Scale for Suicide Ideation; SUV, standardized uptake values.

**Table S5. Sample, treatment, and imaging information of the studies included in the ketamine-induced hypo-functional network analysis**

| **Study** | **Diagnosis** | **Patients**  **N/F** | **Age, years**  **Mean ± SD/**  **Mean (quartiles)** | **Clinical assessment** | | | **Neuroimaging time points** | **Design** | **Application** | **Neuroimaging measure** |
| --- | --- | --- | --- | --- | --- | --- | --- | --- | --- | --- |
|  |  |  |  | **Instrument** | **Pre-ketamine**  **Mean ± SD** | **Post-ketamine**  **Mean ± SD** |  |  |  |  |
| Carlson et al., 2013 [1] | MDD  (DSM-IV) | 20/6 | 47.6 ± 12.2 | MADRS | 33.1 ± 5.7 | 18.7 ± 8.2 (40 min) | Baseline, 120 min | Open label | 0.5 mg/kg over 40 min | CMRGlu |
| Nugent et al., 2014 [13] | BD  (DSM-IV) | 21/15 | 46 ± 12 | MADRS | > 20 | 18 ± 10.5 | 120 min | RCT | 0.5 mg/kg | rMRGlu |
| Li et al., 2016 [3] | MDD  (DSM-IV) | 16/11 | 43.3 ± 11.9 | HDRS-17 | 22.6 ± 5.8 | -32.8% ± 27.2%  (40 min) | Baseline, 40 min | RCT | 0.5 mg/kg over 40 min | SUV of glucose metabolism |
| Reed et al., 2018 [6] | MDD  (DSM-IV) | 33/21 | 36.06 ± 9.74 | MADRS | 33.96 ± 4.64 | 24.78 ± 10.04 | Baseline, 2 d | RCT | 0.5 mg/kg over 40 min | Task-induced activation |
| Chen et al., 2019 [14] | MDD  (DSM-IV) | 16/11 | 43.3 ± 11.9 | MADRS | 33.3 ± 8.6 | 22.3 ± 11.8 | Baseline, 2 d | RCT | 0.5 mg/kg over 40 min | FC |
| Gonzalez et al., 2020 [8] | MDD  (DSM-IV) | 11/3 | 47.7 ± 11.9 | HDRS-17 | 22.44 ± 3.68 | 9.00 ± 7.67 (1 d) | Baseline, 1 h, 6 h, 1 d | Open label | 0.5 mg/kg over 40 min | CBF |
|  |  |  |  | MADRS | 34.89 ± 3.44 | 16.00 ± 12.58 (1 d) |  |  |  |  |
|  |  |  |  | QIDS-SR | 17.78 ± 7.60 | 10.40 ± 6.69 (1 d) |  |  |  |  |
| Zhang et al., 2023 [15] | MDD  (DSM-V) | 16/11 | 37.75 ± 12.63 | MADRS | 27.81 ± 5.72 | 5.50 ± 2.99 | Baseline, 24 h (after 6th infusion, 12 d) | Open label | 0.5 mg/kg over 40 min | FC |
| Wang et al., 2022 [16] | 33MDD 8BD  (DSM-V) | 41/21 | 32 (24, 46.5) | MADRS | 30.90 ± 7.01 | 8.71 ± 5.12 | Baseline, 24 h (after 6th infusion, 12 d) | Open label | 0.5 mg/kg over 40 min | FC |

Abbreviations: BD, bipolar disorder; CBF, cerebral blood flow; CMRGlu, cerebral metabolic rate for glucose; DSM-IV, Diagnostic and Statistical Manual of Mental Disorders, fourth edition; DSM-V, Diagnostic and Statistical Manual of Mental Disorders, fifth edition; FC, functional connectivity; HDRS-17, Hamilton Depression Rating Scale, 17 item; MADRS, Montgomery Asberg Depression Rating Scale; MDD, major depressive disorder; N, number; QIDS-SR, Quick Inventory of Depressive Symptoms, self-report; RCT, randomized controlled trial; rMRGlu, regional metabolic rate of glucose; SUV, standardized uptake values.

**Table S6.** Dice coefficient and statistical significance (*P* values) of the spatial correspondence between our ketamine-induced brain alteration networks and the canonical brain networks revealed by the network correspondence toolbox

| **Canonical network** | **Ketamine-induced hyper-functional network** | | **Ketamine-induced hypo-functional network** | |
| --- | --- | --- | --- | --- |
|  | **Dice coefficient** | ***P* value** | **Dice coefficient** | ***P* value** |
| Visual | 0.0001 | 0.8212 | 0.0696 | 0.3926 |
| Somatomotor | 0.0007 | 0.961 | 0.0374 | 0.7742 |
| Dorsal Attention | 0.0137 | 0.8412 | 0.0067 | 0.9271 |
| Ventral Attention | 0.1456 | 0.2368 | 0.0466 | 0.7423 |
| Limbic | 0.0539 | 0.3606 | 0.2757 | 0.0779^#^ |
| Frontoparietal | 0.1497 | 0.0959^#^ | 0.0009 | 0.999 |
| Default | 0.2882 | 0.009^*^ | 0.208 | 0.0819^#^ |

^*^ *P* < 0.05; ^#^ 0.05 < *P* < 0.1.


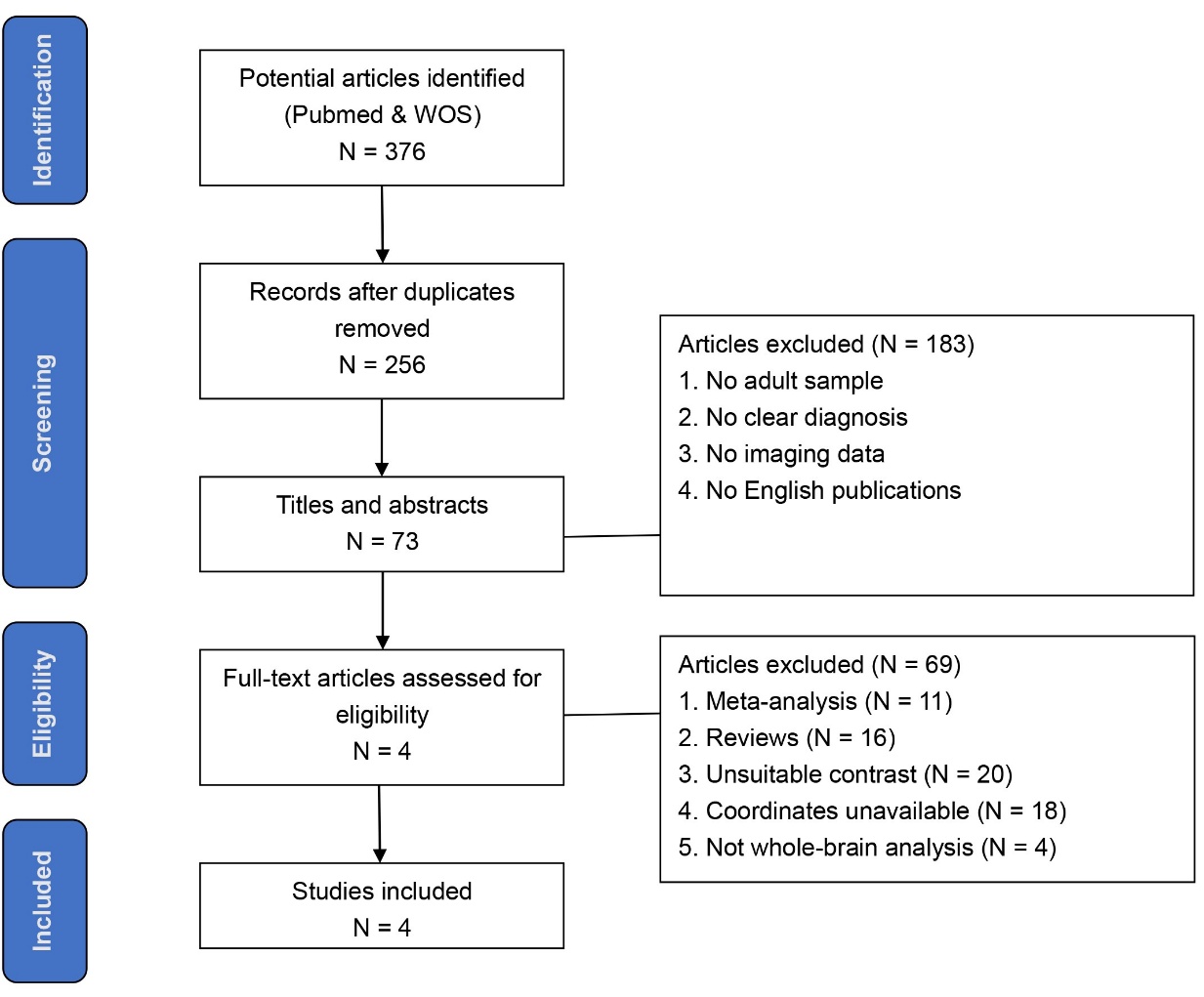


**Figure S1.** A flow diagram of the study selection process.





**Figure S2.** Distributions of the Dice coefficients between the actual ketamine-induced brain functional alteration networks and their null counterparts. **A.** Ketamine-induced hyper-functional network. **B.** Ketamine-induced hypo-functional network.

**
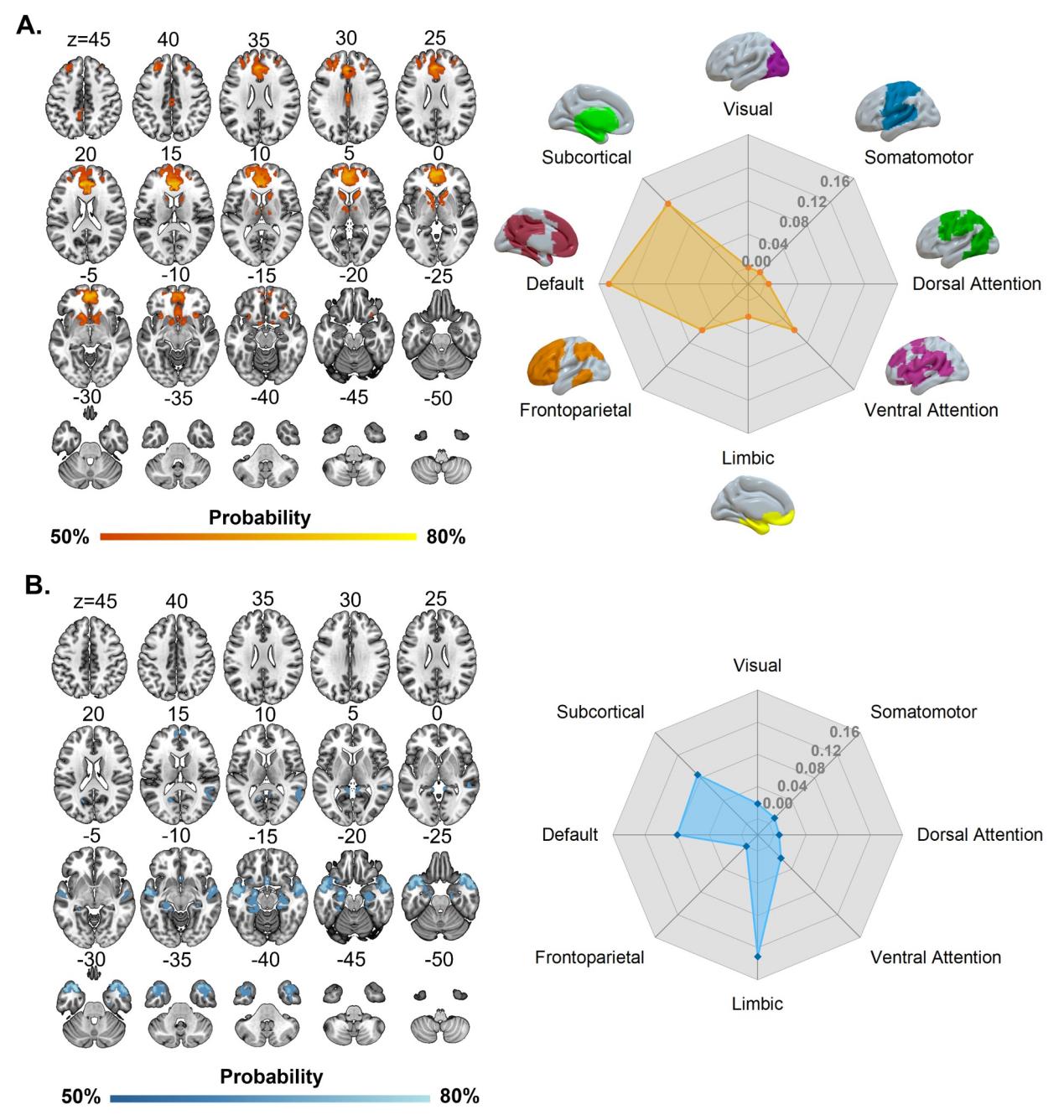
**

**Figure S3.** Ketamine-induced brain functional alteration networks based on 1-mm radius sphere. **A.** Ketamine-induced hyper-functional network (left panel) and its relation to canonical brain networks (right panel). **B.** Ketamine-induced hypo-functional network (left panel) and its relation to canonical brain networks (right panel). Polar plots illustrate the proportion of overlapping voxels between each ketamine-induced brain functional alteration network and a canonical network to all voxels within the corresponding canonical network.

**
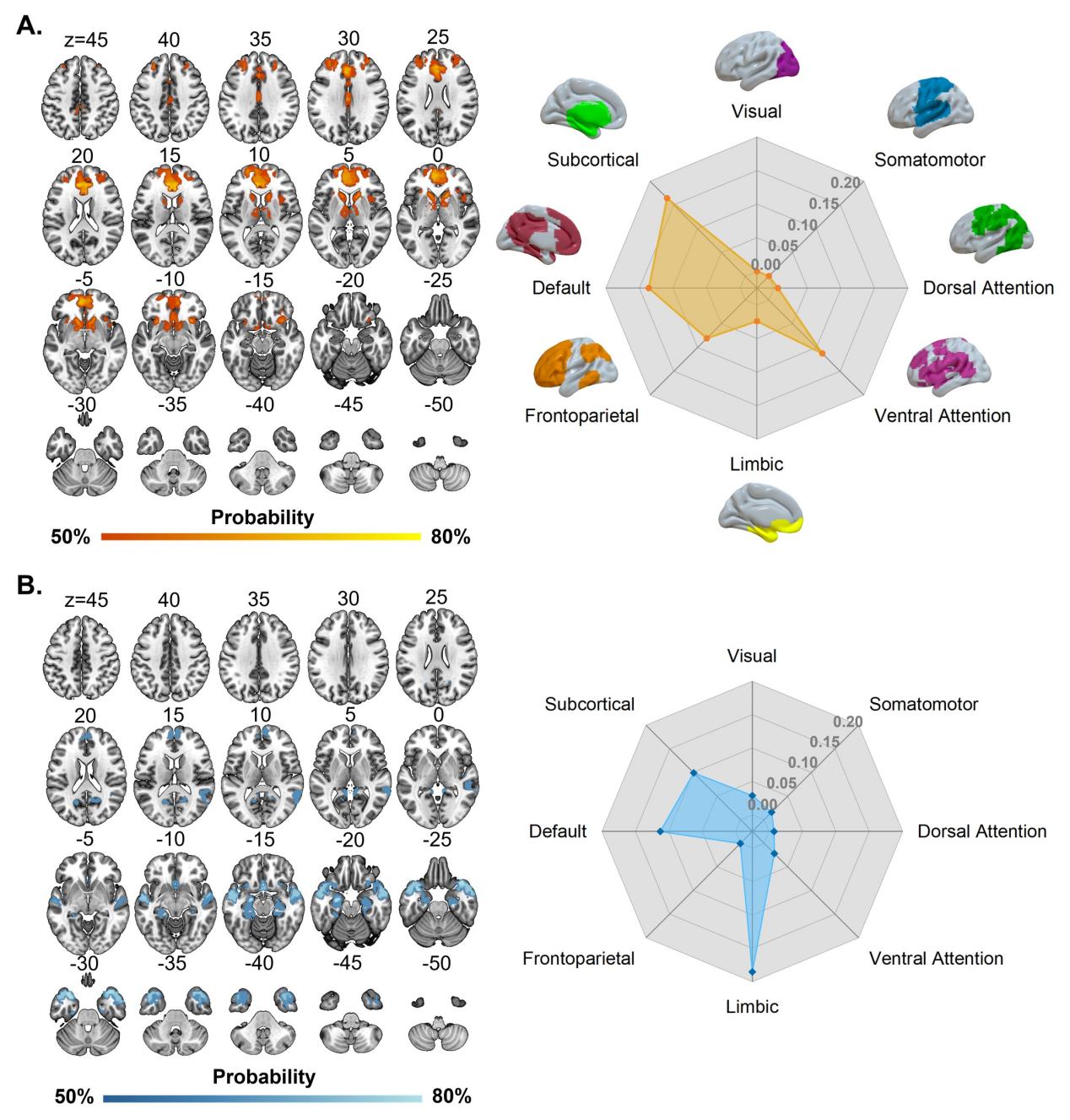
**

**Figure S4.** Ketamine-induced brain functional alteration networks based on 7-mm radius sphere. **A.** Ketamine-induced hyper-functional network (left panel) and its relation to canonical brain networks (right panel). **B.** Ketamine-induced hypo-functional network (left panel) and its relation to canonical brain networks (right panel). Polar plots illustrate the proportion of overlapping voxels between each ketamine-induced brain functional alteration network and a canonical network to all voxels within the corresponding canonical network.


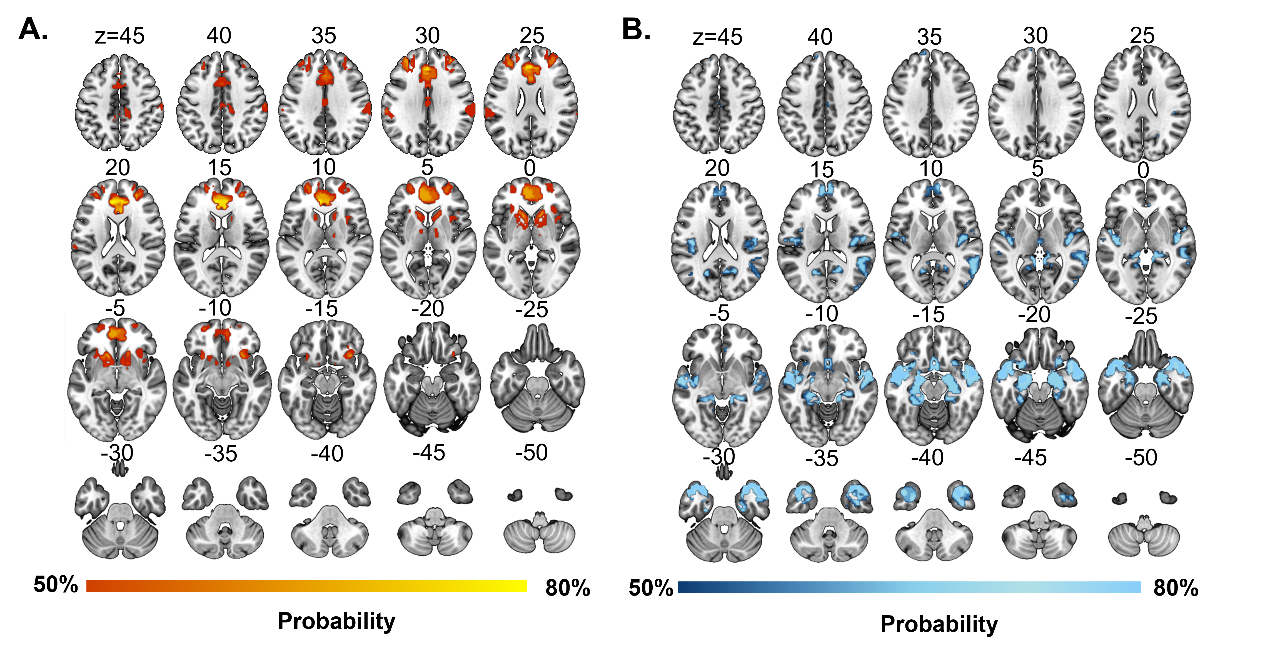


**Figure S5.** Ketamine-induced brain functional alteration networks based on the contrasts focusing on the acute phase of ketamine action (< 2 days). **A.** Ketamine-induced hyper-functional network. **B.** Ketamine-induced hypo-functional network.


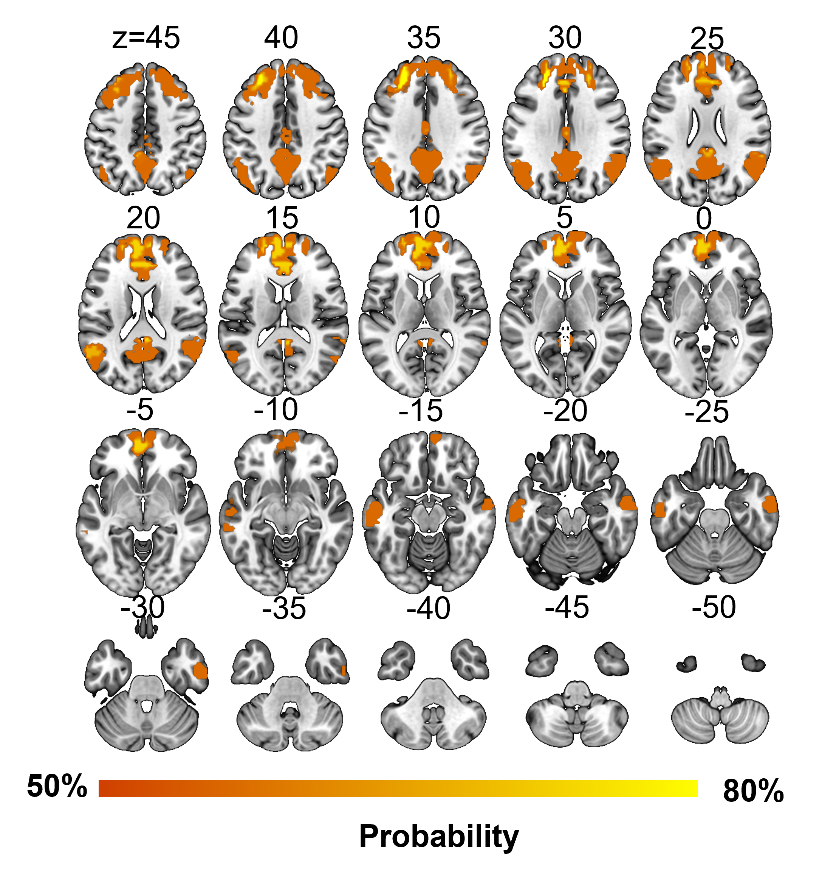


**Figure S6.** Ketamine-induced hyper-functional network derived from resting-state fMRI studies. Abbreviation: fMRI, functional magnetic resonance imaging.

**References**

[1]. Carlson PJ, Diazgranados N, Nugent AC*, et al.* Neural correlates of rapid antidepressant response to ketamine in treatment-resistant unipolar depression: a preliminary positron emission tomography study. *Biol Psychiatry*. 2013 **73:** 1213-1221.

[2]. Murrough JW, Collins KA, Fields J*, et al.* Regulation of neural responses to emotion perception by ketamine in individuals with treatment-resistant major depressive disorder. *Transl Psychiatry*. 2015 **5:** e509.

[3]. Li CT, Chen MH, Lin WC*, et al.* The effects of low-dose ketamine on the prefrontal cortex and amygdala in treatment-resistant depression: A randomized controlled study. *Hum Brain Mapp*. 2016 **37:** 1080-1090.

[4]. Abdallah CG, Averill LA, Collins KA*, et al.* Ketamine Treatment and Global Brain Connectivity in Major Depression. *Neuropsychopharmacology*. 2017 **42:** 1210-1219.

[5]. Abdallah CG, Averill CL, Salas R*, et al.* Prefrontal Connectivity and Glutamate Transmission: Relevance to Depression Pathophysiology and Ketamine Treatment. *Biol Psychiatry Cogn Neurosci Neuroimaging*. 2017 **2:** 566-574.

[6]. Reed JL, Nugent AC, Furey ML, Szczepanik JE, Evans JW, Zarate CA, Jr. Ketamine normalizes brain activity during emotionally valenced attentional processing in depression. *Neuroimage Clin*. 2018 **20:** 92-101.

[7]. Sterpenich V, Vidal S, Hofmeister J*, et al.* Increased Reactivity of the Mesolimbic Reward System after Ketamine Injection in Patients with Treatment-resistant Major Depressive Disorder. *Anesthesiology*. 2019 **130:** 923-935.

[8]. Gonzalez S, Vasavada M, Njau S*, et al.* Acute changes in cerebral blood flow after single-infusion ketamine in major depression: a pilot study. *Neurol Psychiatry Brain Res*. 2020 **38:** 5-11.

[9]. Rivas-Grajales AM, Salas R, Robinson ME, Qi K, Murrough JW, Mathew SJ. Habenula Connectivity and Intravenous Ketamine in Treatment-Resistant Depression. *Int J Neuropsychopharmacol*. 2021 **24:** 383-391.

[10]. Liu H, Wang C, Lan X*, et al.* Functional connectivity of the amygdala and the antidepressant and antisuicidal effects of repeated ketamine infusions in major depressive disorder. *Front Neurosci*. 2023 **17:** 1123797.

[11]. Rengasamy M, Mathew S, Howland R, Griffo A, Panny B, Price R. Neural connectivity moderators and mechanisms of ketamine treatment among treatment-resistant depressed patients: a randomized controlled trial. *EBioMedicine*. 2024 **99:** 104902.

[12]. Chen X, Zhang B, Yuan S*, et al.* Pre-treatment functional connectivity of the cingulate cortex predicts anti-suicidal effects of serial ketamine infusions. *Eur Psychiatry*. 2023 **66:** e31.

[13]. Nugent AC, Diazgranados N, Carlson PJ*, et al.* Neural correlates of rapid antidepressant response to ketamine in bipolar disorder. *Bipolar Disord*. 2014 **16:** 119-128.

[14]. Chen MH, Lin WC, Tu PC*, et al.* Antidepressant and antisuicidal effects of ketamine on the functional connectivity of prefrontal cortex-related circuits in treatment-resistant depression: A double-blind, placebo-controlled, randomized, longitudinal resting fMRI study. *J Affect Disord*. 2019 **259:** 15-20.

[15]. Zhang F, Wang C, Lan X*, et al.* Ketamine-induced hippocampal functional connectivity alterations associated with clinical remission in major depression. *J Affect Disord*. 2023 **325:** 534-541.

[16]. Wang M, Chen X, Hu Y*, et al.* Functional connectivity between the habenula and default mode network and its association with the antidepressant effect of ketamine. *Depress Anxiety*. 2022 **39:** 352-362.
